# Cardiovascular risk factor screening and management of obese patients at an outpatient pediatric cardiology center

**DOI:** 10.1186/s40064-016-3340-9

**Published:** 2016-10-24

**Authors:** Margaret Greco, Arun Sood, Soyang Kwon, Adolfo J. Ariza

**Affiliations:** 1 Pediatrics, Ann & Robert H. Lurie Children’s Hospital of Chicago, 225 E. Chicago Ave., Box 21, Chicago, IL 60611-2605 USA; 2 Pediatrics, Feinberg School of Medicine, Northwestern University, Chicago, IL USA; 3 Rosalind Franklin University, Chicago Medical School, North Chicago, IL USA; 4 Center on Obesity Management and Prevention, Mary Ann & J. Milburn Smith Child Health Research Program, Stanley Manne Children’s Research Institute, Chicago, IL USA

**Keywords:** Children, Pediatric, Congenital heart disease, Obesity, Cardiovascular risk factors, BMI

## Abstract

**Objective:**

To evaluate documentation of cardiovascular (CV) risk factors and obesity management by pediatric cardiologists.

**Study design:**

Review of medical records of obese (≥95th body mass index percentile) 2–17 year-old children presenting to outpatient pediatric cardiology over 1 year. Subjects were categorized as: *heart disease (HD) with increased risk for atherosclerosis*; *HD with average risk for atherosclerosis*; or no HD. Data were evaluated on documentation of the assessment of seven CV risk factors [including recognition of elevated blood pressure (BP)] and management of obesity. Multivariable logistic regression (LR) examined physician documentation of obesity intervention by risk groups, including age and gender.

**Results:**

Data on 730 subjects were analyzed; 16 % had *HD with increased risk for atherosclerosis*, 41 % had *HD with average risk for atherosclerosis*, and 43 % had no HD. Documentation of risk factor assessment was highest for physical inactivity (53 %) and recognition of obesity (47 %). Other factors (child dyslipidemia, diet, dysglycemia, and cigarette exposure) were documented less frequently. Elevated BP was found in 144 patients (20 %); 53/144 (37 %) had documentation of elevated BP recognition. An obesity intervention was documented in 62 % of records and did not significantly differ between risk groups. In the multivariate LR, physician documentation of obesity intervention did not significantly differ between risk groups.

**Conclusions:**

Complete assessment of CV risk factors in obese patients is low. The number of risk factors assessed was similar among patients with *HD with average risk of atherosclerosis* and *HD with increased risk of atherosclerosis*. Increased care coordination between cardiologists and primary care providers may lead to uniform, comprehensive CV risk assessment.

## Background

Cardiovascular disease remains the leading cause of death in adults and is associated with known risk factors including dyslipidemia, hypertension, cigarette smoking, diabetes, obesity, physical inactivity, unhealthy diet and family history of premature coronary artery disease (Roger et al. 2011; Panel 2002; Daniels et al. 2008; Ross 1986). The process of plaque buildup in the arteries, known as atherosclerosis, actually begins in childhood and is thought to be accelerated in certain heart conditions that occur in children. According to the American Heart Association (AHA) Scientific Statement on Cardiovascular Risk Reduction in High-Risk Pediatric Patients (Kavey et al. 2006), the risk of premature atherosclerosis may be increased in heart conditions that involve lesions with coronary artery abnormalities [e.g., Kawasaki disease (Suzuki et al. 2004), repaired transposition of the great arteries (Pedra et al. 2005), anomalous origin of the coronary arteries (Click et al. 1989), transplanted hearts], and obstructive lesions of the left ventricle and aorta [e.g., hypertrophic cardiomyopathy (Hoffman 1978), aortic stenosis (Hoffman 1978), coarctation of the aorta (Strong et al. 1999)].

Obesity can add to the risk of premature atherosclerosis (Berenson et al. 1998) and is often associated with additional cardiovascular disease (CVD) risk factors including poor nutrition/diet, physical inactivity, hyperlipidemia and hypertension (Barlow and The Expert Committee 2007). These CVD risk factors, especially obesity, track from childhood into adult life (Gidding et al. 2004; The et al. 2010). An estimated 17 % of youth (ages 2 through 19 years) has been reported as obese (body mass index [BMI] ≥95th percentile) between 2009 and 2012 (Flegal et al. 2012; Ogden et al. 2012). The prevalence of obesity in children with congenital heart disease has been reported to be as high as 14 % (Pasquali et al. 2009; Pinto et al. 2007). Interventions targeting obesity-associated risk factors may slow the development of atherosclerosis and there is increasing evidence that reduction of these risks delays progression to clinical disease (Expert Panel on Integrated Guidelines for Cardiovascular Health Risk Reduction In Children and Adolescents 2011).

Pediatric cardiology providers have a unique role in the screening for cardiovascular risk factors and prevention of cardiovascular disease. Pinto et al., reported that pediatric cardiologists frequently do not document obesity or weight counseling (Pinto et al. 2007); however, we are not aware of any other studies assessing screening and management practices of a cluster of cardiovascular risk factors by outpatient pediatric cardiology providers. The primary aim of this study is to evaluate the electronic medical record documentation by pediatric cardiology providers of cardiovascular risk factors in obese patients’ ages 2 to 17 years with and without heart disease who presented to an outpatient pediatric cardiology center over a 1 year period. The secondary aim is to evaluate the documentation of an intervention for obesity in those patients.

## Methods

We retrospectively reviewed medical records of obese (BMI ≥95th percentile) children ages 2 to 17 years who presented for an evaluation by a cardiologist at any of our 5 outpatient pediatric cardiology center clinics over a 1 year period. For each subject, the most recent visit between 11/1/10 and 10/31/11 was selected for review. This study was approved by the Ann & Robert H. Lurie Children’s Hospital of Chicago Institutional Review Board (Protocol No. 2013-15193).

We first evaluated all age-eligible visits in the study period to identify potential visits that met the BMI criteria. We excluded visits with: missing height or weight data; if the height measurement documented was inconsistent with past height measurements (i.e., shorter than prior visit); if the height measurement documented had extreme height z-score (≥3 or ≤−3); if the patient had Down syndrome; or if the visit was performed at a location other than an outpatient pediatric cardiology center clinic. During the study period, there were 5491 patients ages 2–17 years presenting for care, of which 730 (13 %) were obese and met study eligibility criteria.

Data collected via download from the electronic medical record (EMR) included socio-demographics, anthropometrics, blood pressure (BP) measurements, and visit diagnoses. These data were used to identify types of heart conditions and surgical interventions for each subject, which were then verified by individual review of the record. Visit summary letters to the referring provider were individually reviewed to assess documentation related to eight key cardiovascular risk factors. The CVD risk factors assessed [child dyslipidemia, elevated blood pressure, cigarette smoke exposure, dysglycemia, obesity, physical inactivity, and unhealthy diet; and family history of premature coronary artery disease (CAD)] were chosen based on those noted in Third Report of the National Cholesterol Education Program (NCEP) Expert Panel on Detection, Evaluation, and Treatment of High Blood Cholesterol in Adults (Panel NC 2002). Elevated BP was defined as systolic and/or diastolic BP ≥95th percentile based on the Fourth Report of the National High Blood Pressure Education Program (National High Blood Pressure Education Program Working Group on High Blood Pressure in Children and Adolescents 2004). BP data were one-time measurements obtained with either a Dinamap automated BP monitor or by manual sphygmomanometer. Since all patients had BMI and BP documented automatically, we specifically assessed for *recognition* of obesity status and *recognition* of elevated BP as documented in the letter to the referring provider. Table 1 describes the criteria used to determine if the cardiovascular risk factor was assessed during the visit and if obesity management was documented.

**Table 1:** Definitions used to assess for cardiovascular risk factor documentation and obesity management

Risk factor	Definition^a^
Obesity	Documentation of BMI value plus documentation of interpretation “obese”, “quite heavy”, “elevated BMI”, “BMI is high”, “BMI is ≥95th percentile”, “large for age”, or “overweight” (this term was sometimes used by clinicians when the patient was at or above the 95th percentile)
Dysglycemia	Documentation of glucose-related labs (e.g., glucose, hemoglobin A1c, insulin, or liver function tests) or exam findings of acanthosis nigricans
Physical inactivity	Documentation of screen time, participation in sports, and/or participation in other physical activities
Child dyslipidemia	Documentation of lipid-related labs (e.g., lipid panel, cholesterol, triglycerides) or recommendation to test for dyslipidemia
Elevated blood pressure	Documentation of elevated blood pressure such as “high blood pressure” or “hypertension”
Unhealthy diet	Documentation of dietary habits
Cigarette smoke exposure	Documentation of caregiver smoking status or exposure to tobacco in the home or daycare
Family history of CAD	Documentation of positive or negative family history of early CAD
Obesity management	Documentation of recommendations given on diet or exercise such as encouraging a “heart healthy lifestyle”, “weight reduction”, “60 min exercise/day’’ or “daily aerobic activity’’; documentation of obesity associated lab evaluation (e.g., glucose, lipid panel or LFTs); documentation of follow-up for obesity or referral to an obesity related subspecialist (lipid, nutrition, obesity clinic)

*BMI* body mass index; *CAD* coronary artery disease; *LFTs* liver function tests

^a^Risk factor recorded as documented if any of the above definitions were noted in the letter to the referring provider

### Ethical considerations

This study was approved by our Institutional Review Board and is in compliance with all ethical considerations.

### Analysis

BMI percentiles and height z-scores were interpreted using Epi Info 3.5.3 (National Center for Health Statistics, Centers for Disease Control & Prevention, CDC, Atlanta, GA, 2011) using the CDC 2000 growth references (Kuczmarski et al. 2002). Blood pressure percentiles were interpreted using Health Indicators Analyzer (HIA) software (©2003, Ann & Robert H. Lurie Children’s Hospital of Chicago, Chicago, IL), which uses gender-, age-, height-specific NHLBI references (National High Blood Pressure Education Program Working Group on High Blood Pressure in Children and Adolescents 2004).

We first grouped subjects into three categories based on levels of risk for atherosclerosis described by the AHA Scientific Statement (Kavey et al. 2006): (1) obese patients with *HD with increased risk for atherosclerosis* (e.g., heart transplant, Kawasaki disease, anomalous origin of the coronary arteries, transposition of the great arteries, coarctation of the aorta, aortic stenosis, or hypertrophic cardiomyopathy); (2) obese patients with *HD with average risk for atherosclerosis* (e.g., patients with a cardiac diagnosis excluding those listed above); and (3) obese patients with no HD (e.g., patients referred to the cardiology clinic for evaluation of chest pain, palpitations, murmur or family history of heart disease, etc., but found to have no organic heart disease as documented in the letter to the referring provider).

Frequency analyses of the documentation of the assessment of cardiovascular risk factors and the management of obesity were performed for the total sample and by group. Chi square analyses were used to examine documentation of the assessment of the CV risk factors by risk for atherosclerosis groups; to adjust for multiple CV risk factors evaluated, significance was set at p < 0.01 for these analyses.

Chi square analyses and one-way ANOVA were conducted to determine demographic and BMI-related differences by risk for atherosclerosis group, as appropriate. A multivariable logistic regression model was used to assess physician documentation of recognition of obesity (dependent variable) by risk for atherosclerosis group. Variables significant (p < 0.05) in univariate analyses (child age group and gender) were included as independent variables in the model; however, due to missing data, race/ethnicity was not included. Data were analyzed using SPSS statistics (Version 20.0.0, IBM Corp., Armonk, NY).

## Results

The 730 obese subjects were mean age 10.4 years (SD 4.4 years) and had mean BMI 26.6 kg/m^2^ (SD 6.1 kg/m^2^; range 17.9 kg/m^2^ to 51.0 kg/m^2^). Baseline characteristics of the total study population and subgroups are shown in Table 2. The subgroups differed significantly in age and gender.

**Table 2:** Baseline characteristics of the total study population and subgroup

Characteristics	Total n = 730	Atherosclerosis risk group			p value
		No heart disease n = 313 (43 %)	Heart disease with average risk n = 297 (41 %)	Heart disease with increased risk n = 120 (16 %)	
Age, years, mean (SD)	10.4 (4.4)	10.9 (4.2)	9.9 (4.5)	10.4 (4.5)	0.014
Age groups, n (%) (years)					0.020
12–17	302 (41)	143 (46)	110 (37)	49 (41)	
7–11	247 (34)	109 (35)	100 (34)	38 (32)	
2–6	181 (25)	61 (19)	87 (29)	33 (28)	
Gender, n (%)					0.023
Male	462 (63)	195 (62)	178 (60)	89 (74)	
Female	270 (37)	118 (38)	119 (40)	31 (26)	
BMI, kg/m^2^, mean (SD)	26.6 (6.1)	26.9 (6.2)	26.1 (6.0)	26.7 (6.2)	0.243
BMI Z-score, mean (SD)	2.1 (0.4)	2.1 (0.4)	2.1 (0.4)	2.1 (0.4)	0.165
Race/ethnicity, n (%) (n = 681)					0.016
Hispanic	325 (45)	158 (50)	113 (38)	54 (45)	
White	251 (34)	94 (30)	117 (39)	40 (33)	
African American	105 (14)	36 (12)	47 (16)	22 (18)	

*BMI* body mass index

The most common heart conditions in the 120 obese patients with *HD with increased risk for atherosclerosis* were: aortic stenosis (38 %), anomalous coronary origin (14 %) and transposition of the great arteries (13 %). The most common heart conditions in the 297 obese patients with *HD with average risk of atherosclerosis* were: supraventricular tachycardia 38 (17 %), pulmonary stenosis 30 (10 %), and bicuspid aortic valve without aortic stenosis 29 (10 %). The most common conditions found in obese patients with no HD were murmur (32 %), chest pain (30 %), and vasovagal syncope (10 %). Among children with diagnosed heart disease, 46 % of those with *HD with increased risk of atherosclerosis* and 29 % of those with *HD with average risk of atherosclerosis* had received surgical- or catheter-based interventions. Documentation of exercise restrictions among children with diagnosed HD was noted for 25 % of those with *HD with increased risk of atherosclerosis* and 12 % with *HD with average risk of atherosclerosis*.

### Documentation of cardiovascular risk factor screening

**Fig. 1:**
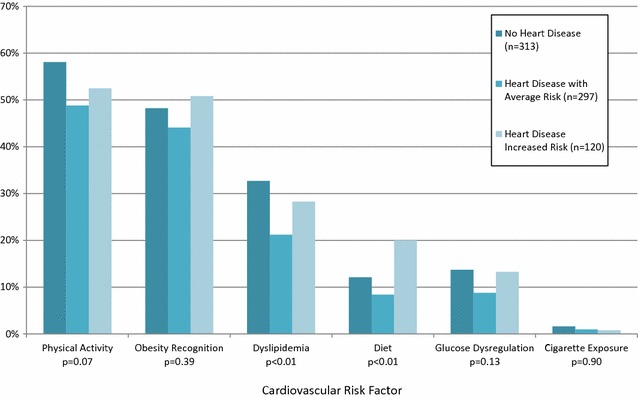
Documentation of the assessment of cardiovascular risk factors by subgroup

All patients had weight and height measured and BMI recorded and plotted on the EMR growth record. The BMI chart showed a plot at or greater than the BMI 95th percentile. However, obesity was documented as recognized in 47 % (343/730) of the records. Blood pressure was measured for all subjects and 20 % (144/730) had elevated BP; however, elevated BP was documented as recognized in 37 % (53/144). Documentation of child physical inactivity assessment of was found in 53 % of all records, dyslipidemia in 27 %, diet in 12 %, dysglycemia in 12 %, and cigarette exposure in 1 %. The family history of premature CAD was documented as “unchanged from previous” in 27 % (195/730) of medical records, therefore, an accurate assessment of documentation of this risk factor could not be obtained. A detailed documentation of cardiovascular risk factor assessment by risk factor group is displayed in Fig. 1.

There was no statistical difference for documentation of the assessment of the cardiovascular risk factors studied among the three groups except for the assessment of dyslipidemia and diet. The assessment of dyslipidemia was most frequently documented among patients with no HD (33 %), followed by patients with *HD with increased risk of atherosclerosis* (28 %) and patients with *HD with average risk of atherosclerosis* (21 %), (p < 0.01). The assessment of diet was most frequently documented in patients with *HD with increased risk for atherosclerosis* (20 %), followed by patients with no HD (12 %) and patients with *HD with average risk for atherosclerosis* (8 %), (p < 0.01).

In the multivariate LR there was no statistically significant differences in the recognition of obesity between those with no HD and those with *HD with average risk of atherosclerosis* [adjusted odds ratio (aOR) 1.0], (95 % confidence interval [CI] 0.7–1.4), or those with *HD with increased risk of atherosclerosis*, aOR 1.3 (95 % CI 0.8–2.1). Compared to children 2–6 years of age obesity was more often recognized in patients 7–11 years (OR 4.0, 95 % CI 2.5–6.2) and children 11–17 years (OR 7.5, 95 % CI 4.8–11.7). There was no significant difference in obesity recognition by gender.

### Documentation of an intervention for obesity

Our definition of an intervention for obesity included documentation of counseling related to diet or activity, related referrals and related laboratory testing (Table 1). Based on this comprehensive definition, an intervention for obesity was documented in 62 % of records. The most frequently documented intervention was physical activity counseling (34 %), followed by diet counseling (29 %). Documentation of an intervention for obesity did not vary significantly by risk for atherosclerosis group (p = 0.250). An intervention for obesity was most frequently documented in patients aged 12–17 (54 %), followed by patients aged 7–11 (35 %) and patients aged 2–6 years (11 %), (p < 0.001).

## Discussion

This study provides information on cardiovascular risk factor screening and management practices of obese patients seen by outpatient cardiologists. Data abstraction for the study finished 1 year before publication of the NHLBI pediatric cardiovascular risk prevention guidelines. This study presents baseline information on the documentation of risk factors prior to the publication of the guidelines in 2011 (Expert Panel on Integrated Guidelines for Cardiovascular Health Risk Reduction In Children and Adolescents 2011). Many of these patients, especially those having heart disease associated with risk of accelerated atherosclerosis, are at high risk for developing cardiovascular disease and our study shows that there is room for improvement in the documentation of assessment and counseling on CVD risk factors in obese patients.

The recognition of obesity was documented in less than half of the records of the obese patients reviewed and there were no significant differences in obesity recognition documentation by risk of atherosclerosis group. Using our definition of an intervention for obesity, we found that documentation of an intervention for obesity was higher than the documentation of the recognition of obesity, which suggests that more providers may be recognizing obesity than was documented or that providers maybe documenting diet and activity counseling as a routine practice. Pediatric cardiology clinicians were more likely to document an intervention for obesity in older children (≥12 years of age).

Due to the growing realization that cardiovascular risk begins in childhood, the importance of obesity as a component of cardiovascular risks and the strong tracking of obesity status from child through adult years (Strong et al. 1999; Berenson et al. 1998; The et al. 2010), assessment and counseling to promote heart-healthy habits and healthy weight status is becoming an important component of well child care. Incorporating preventive cardiology into cardiologist routine care is needed to reinforce these primary care counseling efforts. As experts in cardiovascular health, pediatric cardiology clinicians play a unique role in conveying the adverse cardiovascular effects associated with obesity and other factors raising cardiovascular risks to patients and their families. In addition, these adverse effects can be compounded in patients with underlying heart disease.

It has been shown in previous studies that obese and overweight children have higher systolic and diastolic BP (May et al. 2012). In our study, 20 % of patients had elevated systolic and/or diastolic BP. This was documented as recognized in one-third of those patients. Hypertension is an important comorbidity of obesity with negative cardiovascular effects. As the obesity epidemic continues, pediatric cardiology clinicians can play an important role in the screening and recognition of elevated BP.

Moving forward, the NHLBI pediatric cardiovascular risk prevention guidelines provide a framework on which to build improved care. Developing new strategies that would facilitate routine cardiovascular risk factor prevention seems warranted. A possible option would be to develop an electronic medical record-based solution such as the implementation of specific cardiovascular risk factor assessment and counseling prompts, and/or the implementation of EMR “smart sets” to facilitate routine implementation of preventive guidelines. Further research is needed on evaluating the impact of implementing care intervention strategies on actual clinical care and on the health impact of the implementation of such strategies.

### Study limitations

There are several limitations to this study. First, this is a retrospective medical record review. Data on cardiovascular risk factor screening, obesity recognition and obesity management were only obtained from the documentation in the visit note and clinic letter to the referring provider for a single visit. It might be possible that the factors studied were addressed during the visit but there was no documentation of such actions. Additional information related to obesity and cardiovascular risk factor screening may have been discussed during the office visit or at prior visits. Finally, data collected were limited to a single institution and may not reflect the overall cardiovascular screening and management practices of pediatric cardiology clinicians.

## Conclusions

The documentation of the assessment of CV risk factors in obese patients varies by risk factor. Complete assessment of CV risk factors is low. The number of risk factors assessed was similar for patients with *HD with average risk of atherosclerosis* and *HD with increased risk of atherosclerosis*. Strategies to increase care coordination among pediatric cardiologists and primary care providers may be needed to ensure uniform, comprehensive CV risk assessment.

## Abbreviations

BMI: body mass index
BP: blood pressure
HD: heart disease
